# Supplementary material for: Cationic Antimicrobial Copolymers Reveal Immunomodulatory Properties in Lipopolysaccharide Stimulated Macrophages in Vitro
Source: Biomacromolecules. 2025 Oct 10;26(11):7789–801. doi: 10.1021/acs.biomac.5c01280 (PMC12606633; doi:10.1021/acs.biomac.5c01280)
Supplement: Supplementary file 1 [file bm5c01280_si_001.pdf]

Supplementary Information

Cationic Antimicrobial Copolymers Reveal Immunomodulatory Properties in Lipopolysaccharide Stimulated Macrophages *in Vitro*

Sophie Laroque[a], Jim Harris[d,e], Santhosh Kalash Rajendrakumar[a], Vadim Vasilyev[b], Jaspreet Grewal[b], Robert Dallmann[b], Katherine Locock[d], Sébastien Perrier[a,b,c]

[a] *Department of Chemistry, University of Warwick, Gibbet Hill Road, Coventry CV4 7AL, UK.*

[b] *Division of Biomedical Science, Warwick Medical School, University of Warwick, Coventry, CV4 7AL, UK*

[c] *Faculty of Pharmacy and Pharmaceutical Sciences, Monash University, 381 Royal Parade, Parkville, VIC 3052, Australia*

[d] *CSIRO Manufacturing, Clayton, Victoria 3168, Australia*

[e] *School of Clinical Sciences at Monash Health, Faculty of Medicine, Nursing and Health Sciences, Monash University, Clayton, Victoria 3168, Australia*

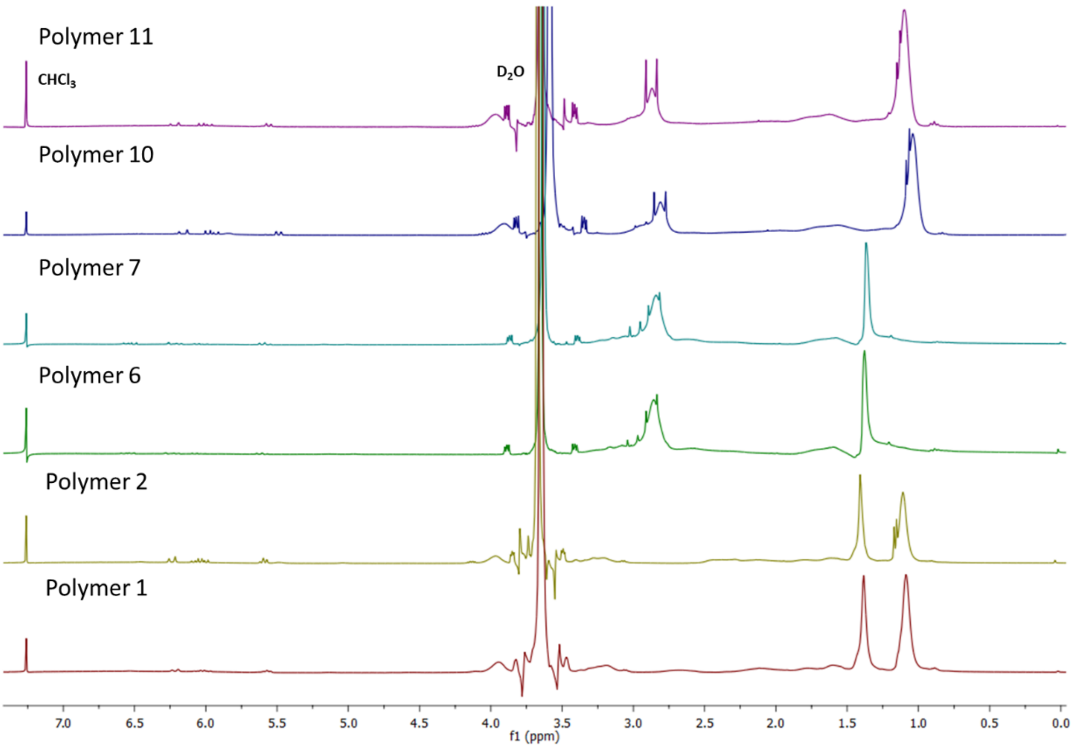

Figure S1: ^{1}H-NMR Spectra of statistical copolymers ($CDCl_3$, 400 MHz).

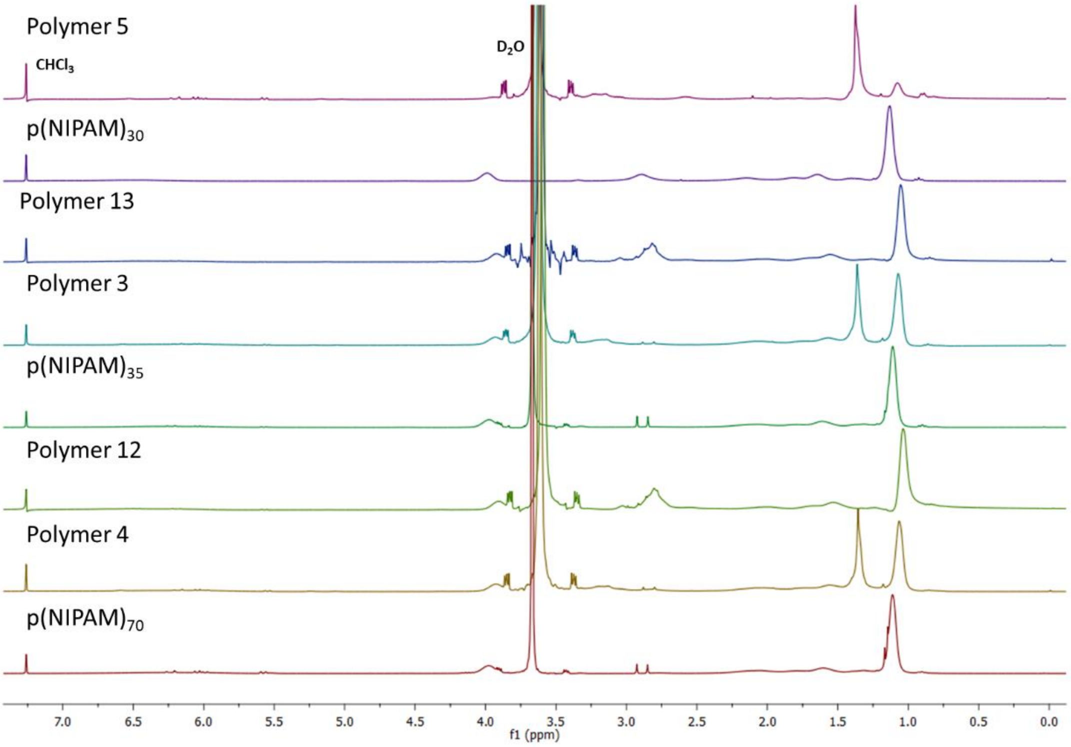

Figure S2: ^{1}H-NMR Spectra of diblock copolymers with pNIPAm$_x$ block ($CDCl_3$, 400 MHz).

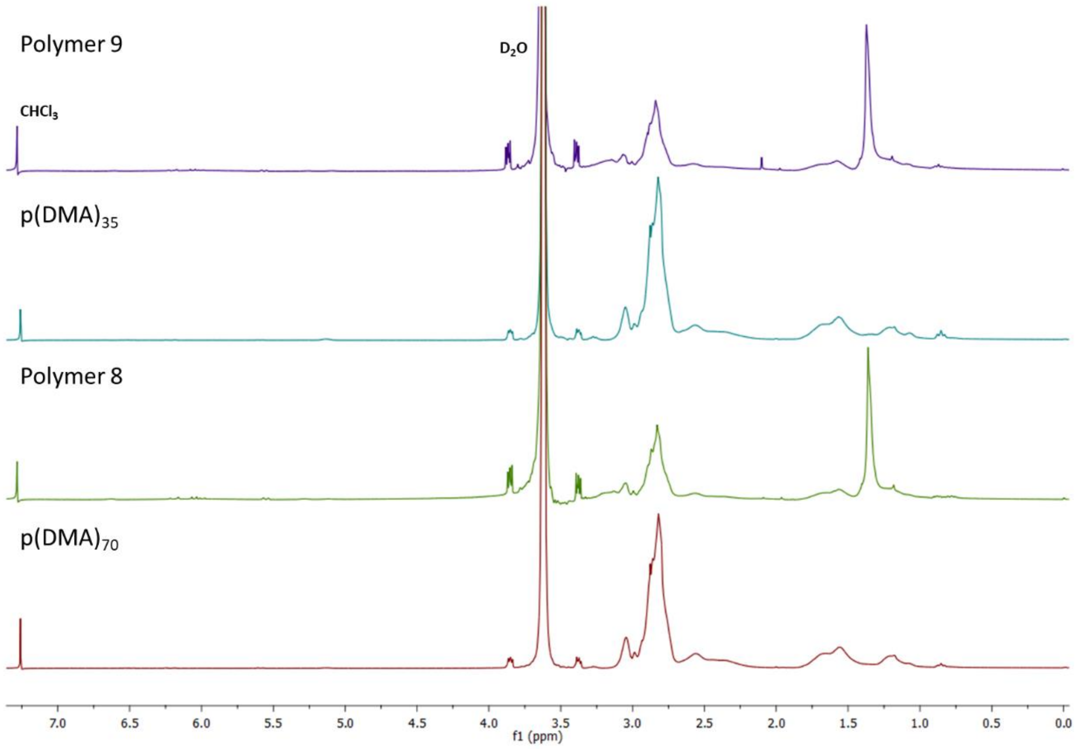

Figure S3: ^{1}H-NMR Spectra of diblock copolymers with pDMA$_x$ block ($CDCl_3$, 400 MHz).

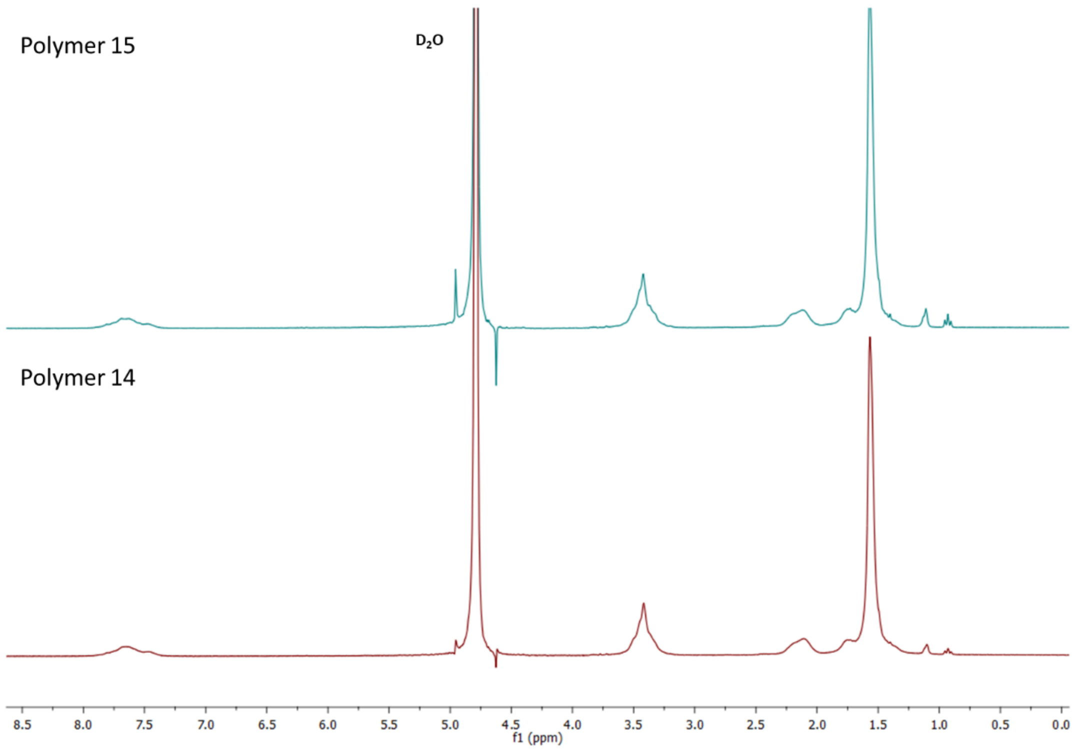

Figure S4: ^{1}H-NMR Spectra of pAMPs homopolymers (D_2O, 400 MHz).

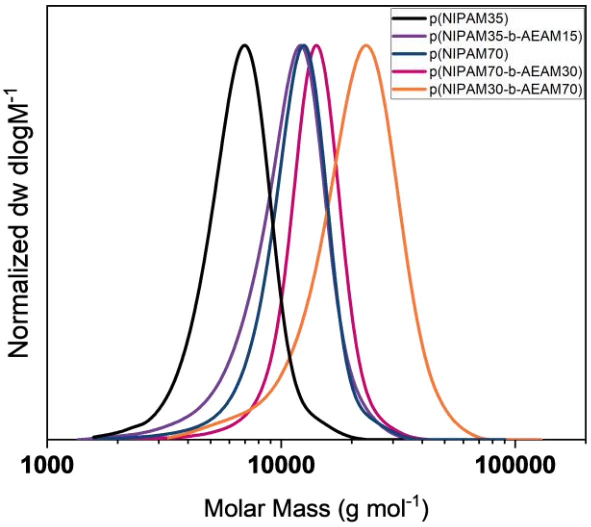

Figure S5: GPC traces of chain extensions for diblock copolymers p(NIPAm$_x$-*b*-BocAEAM$_x$) (PMMA standard, DMF).

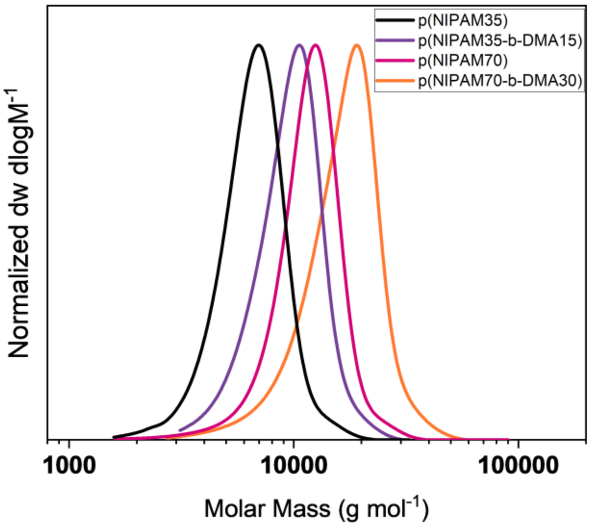

Figure S6: GPC traces of chain extensions for diblock copolymers p(NIPAm$_x$-*b*-DMA$_x$) (PMMA standard, DMF).

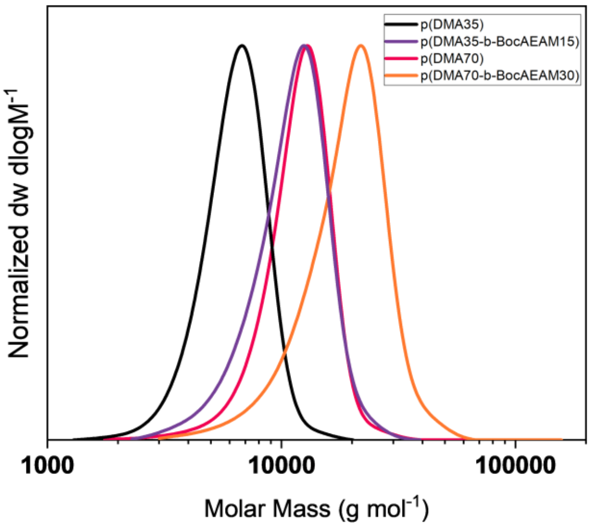

Figure S7: GPC traces of chain extensions for diblock copolymers p(DMA_x-*b*-$BocAEAm_x$) (PMMA standard, DMF).

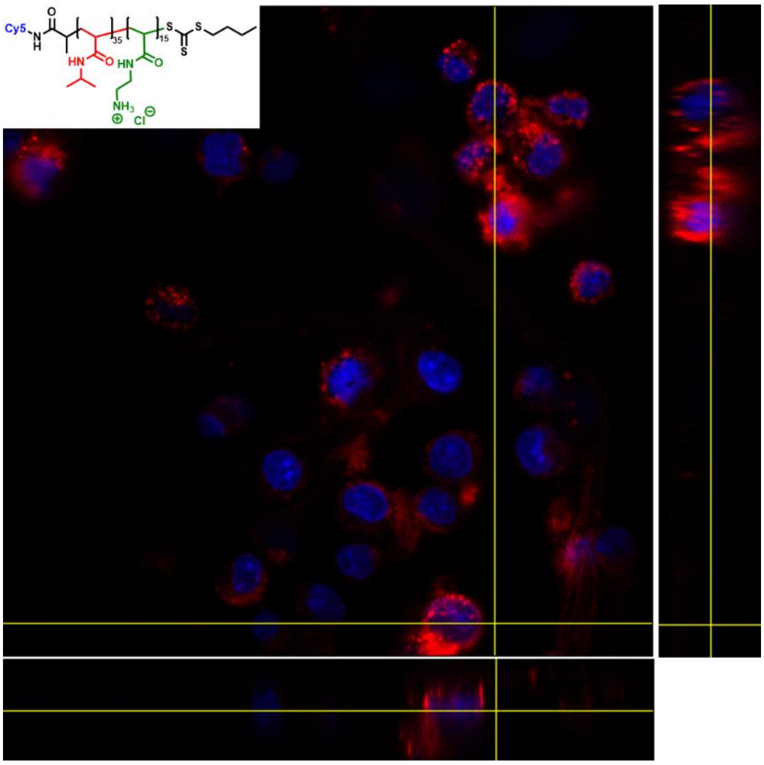

Figure S8: Z plane of confocal microscopy image of RAW 264.7 cells treated with Cy5-functionalised diblock copolymers 3 / p(NIPAm$_{35}$-*b*-AEAm$_{15}$).

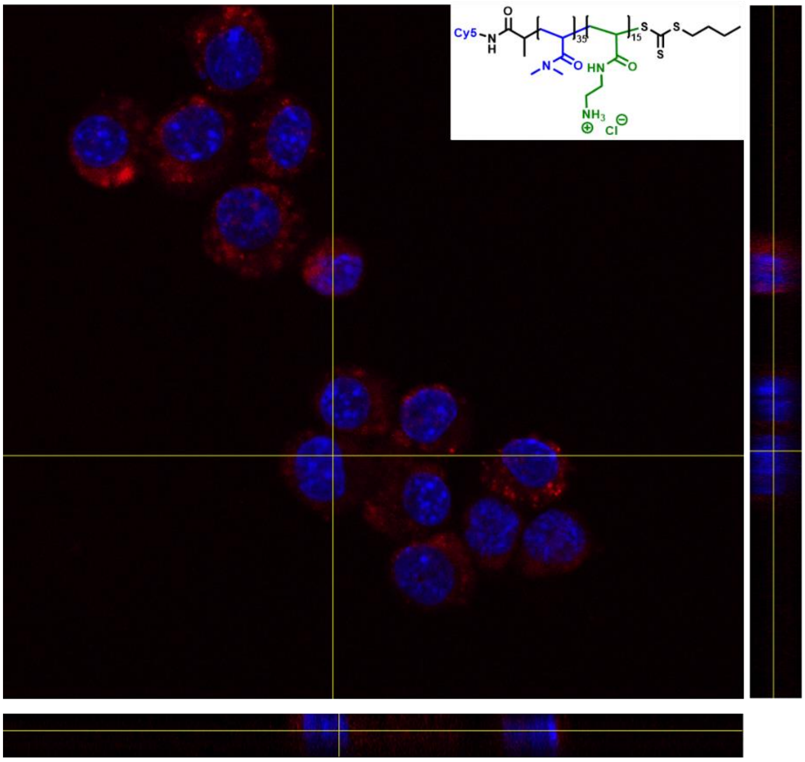

Figure S9: Z plane of confocal microscopy image of RAW 264.7 cells treated with Cy5-functionalised diblock copolymers 8 / p(DMA$_{35}$-*b*-AEAm$_{15}$).

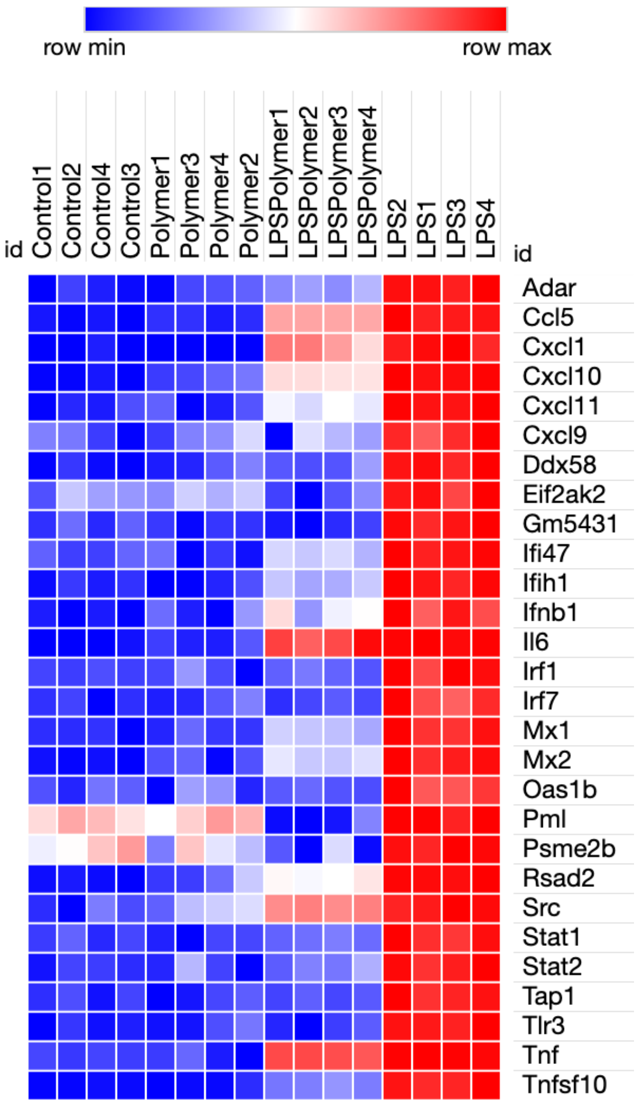

Figure S10: Heatmap of differentially expressed genes related to LPS induced inflammation.

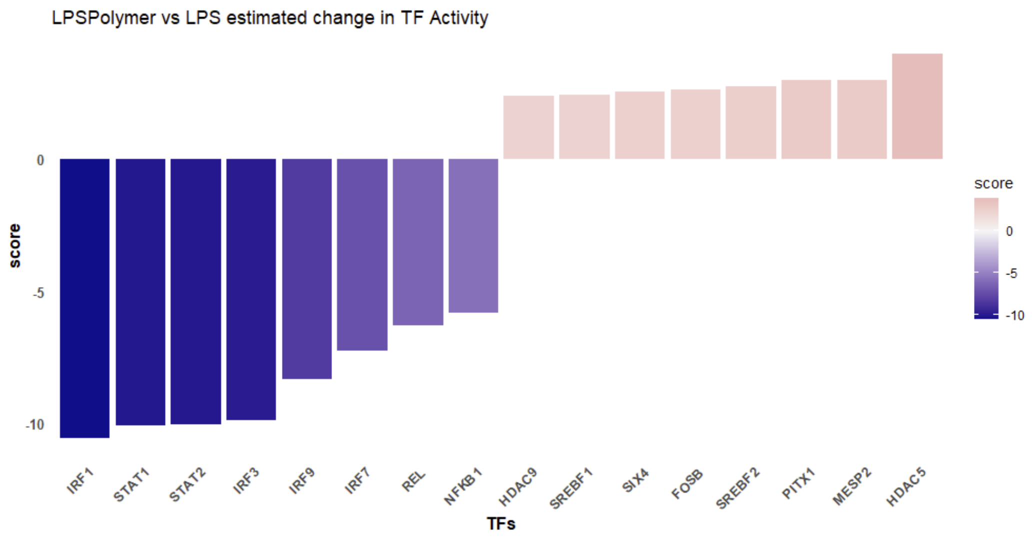

Figure S11: The top 16 altered transcription factors (TF) altered for LPS /polymer Vs LPS only.

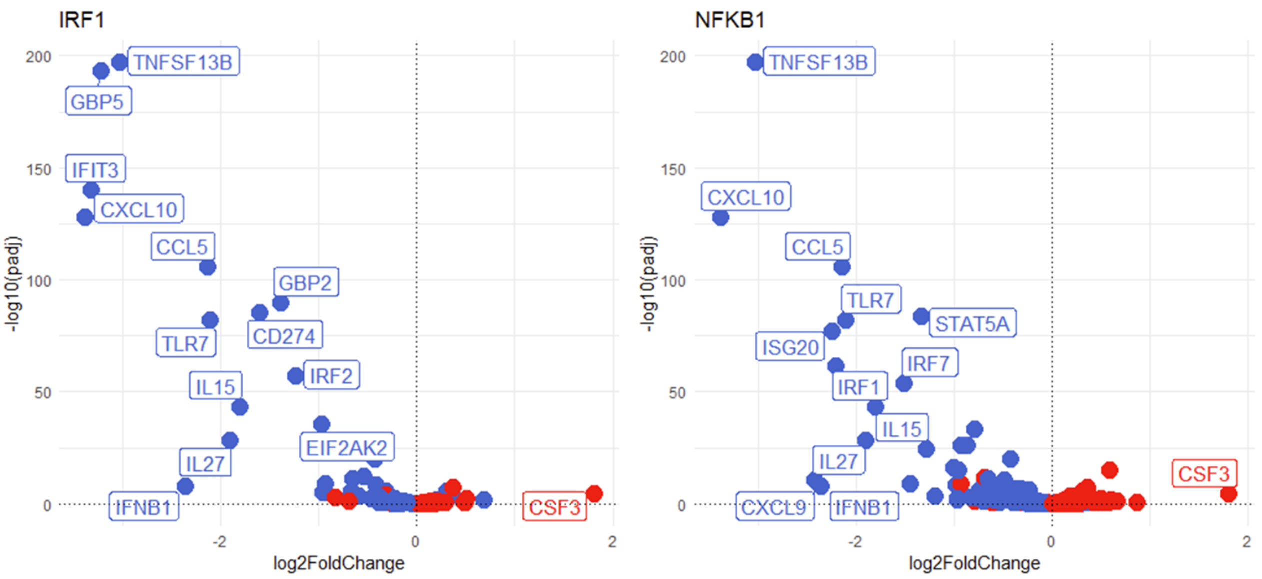

Figure S12: Volcano plot of TFs i.e., IRF and NFκB for LPS /polymer Vs LPS only.
